# Bronchodilatory Effect of Hydrogen Sulfide in Rat

**Published:** 2012

**Authors:** Mohammad Kazem Gharib-Naseri, Shadan Saberi, Seyyed Ali Mard, Seyyed Mahmood Latifi

**Affiliations:** 1 *Physiology Research Centre and Department of Physiology, School of Medicine, Ahvaz Jundishapur University of Medical Sciences, Ahvaz, Iran *; 2 *School of Health, Ahvaz Jundishapur University of Medical Sciences, Ahvaz, Iran *

**Keywords:** Bronchodilator, Hydrogen sulfate, Rat

## Abstract

**Objective(s):**The aims of present study were to elucidate the effect of NaHS as a H_2_S donor on precontracted rat trachea smooth muscle, role of epithelium and nitric oxide in this action.

**Materials and Methods:**Tracheal rings from male adult Wistar rats were isolated and mounted in an organ bath containing Krebs–Henseleit solution under 1.5 g resting tension and contractions were recorded isometrically. After equilibrium period (60 min), cumulative concentrations of NaHS (0.2-1.2 mM) were applied on the tracheal basal tone or on the plateau of contractions induced by KCl (60 mM) or carbachol (CCh, 0.55 μM) in the absence and presence of certain antagonists and inhibitors.

**Results:**The tracheal basal tone was unaffected by NaHS but tracheal contractions induced by KCl and CCh were attenuated by NaHS in a concentration-dependent manner (*P*< 0.001). Removing the tracheal epithelial did not attenuate the NaHS spasmolytic effect in the tissue precontracted by KCl and CCh. The bronchodilatory effect was unaffected by tissue incubation (30 min, 1 μM) with, glibenclamide, propranolol, indomethacin, methylene blue (10 μM), and L-NAME (300 μM).

**Conclusion:**It seems that bronchodilatory effect of H_2_S was not mediated by K_ATP_ channels, β-adrenoceptors, epithelium and production of nitric oxide, cGMP and prostaglandins. Since CCh and KCl activate Ca^2+^ influx and CCh promotes Ca^2+^ from intracellular pool as well, therefore, we may conclude that the relaxant effect of NaHS was mediated by the Ca^2+^ influx blockade and cholinergic receptors inactivation. This preliminary study shows the possible therapeutical property of H_2_S in obstructive pulmonary diseases.

## Introduction

Hydrogen sulfide (H_2_S) with the smell of rotten egg is an irritant gas but with some biological effects and it has been described as third gaseous transmitter (1). H_2_S is a chemical hazard in certain manufacturing industries (2) but recently it has been known as a non-waste material (3). Hydrogen sulfide is produced in the intestine by sulfate-reducing bacteria during colonic fermentation (4) and it has been suggested that H_2_S is produced by red blood cells and vascular smooth muscle cells (5) and can be involved in the etiopathogenesis of chronic diseases such as ulcerative colitis (6).

Hydrogen sulfide causes inflammation in the airway (2). Endogenous H_2_S has an important regulatory influence on pulmonary collagen remodeling (7) and inducing hypotension by direct inhibition of angiotensin-converting enzyme (ACE), vasorelaxation, and inhibition of bradykinin degradation (8). On the other hand, it is reported that H_2_S reducing rat airway inflammation induced by ovalbumin and even suggested that H_2_S can be a novel target in prevention and treatment of asthma (9). Hydrogen sulfide induces contraction in some smooth muscle cell such as in guinea-pig airways (2). It has been shown that endogenous H_2_S concentration was increased in patients with stable chronic obstructive pulmonary disease (9). In central nervous, gastrointestinal and vascular systems the enzymes cystathionine β-synthase (CBS), cystathionine γ-lyase (CSE) (10) and 3-mercaptopyruvate sulfutransferase (3MST) (11) are involved respectively and they use L-cystein as a major substrate. The spasmogenic effect of NaHS on guinea-pig trachea (2) and relaxant activity in mouse gastric fundus smooth muscle (12) have been reported. Because of this controversy, our aim in the present study was to investigate the effect of NaHS as a H_2_S donor on the rat trachea smooth muscle and to reveal its underlying mechanism(s).

## Materials and Methods

***Reagents***

Carbachol (CCh), N^ω^-nitro-L-arginine methyl ester (L-NAME), glibenclamide, NaHS, indomethacin, and propranolol were purchased from Sigma (USA), methylene blue and mineral solutes purchased from Merck (Germany). 

***Animals and tissue preparation***

All animal experiments were approved by the Animal Ethics Committee of the Ahvaz Jundishapur University of Medical Sciences (AJUMS) and carried out in accordance with the AJUMS guideline for animal research. Male adult Wistar rats (222.5±3.1 g) were purchased from Animal Facility of AJUMS and were housed in cages with ambient temperature at 20-24 ºC, 12 hr light/dark cycle and free access to water and food. The rats were anaesthetized by diethyl ether and their trachea was dissected out. In a cold oxygenated Krebs-Henseleit solution the connective tissue was removed. A piece of trachea (4-5 mm) with 5-6 cartilage rings was cut and mounted between two stainless steel hooks horizontally. The lower hook was fixed at the bottom of the organ bath and upper one was connected to an isometric transducer (UF1 Harvard transducer, UK) connected to an ink writing curvilinear recorder (Harvard Universal Oscillograph, UK). The organ bath (10 ml) contained Krebs-Henseleit (pH 7.4, 37 ºC) with following composition (mM): NaCl (118), KCl (4.7), CaCl_2 _(2.52), MgSO_4 _(1.64), KH_2_PO_4 _(1.18), NaHCO_3 _(7) and glucose (5.5), bubbled with oxygen. Tissue was then maintained under 1.5 g initial tension and allowed to equilibrate for 60 min during which bath solution was refreshed every 15 min and resting tension (1.5 g) was readjusted.

After equilibrium period, the trachea was contracted by 60 mM of KCl or by 0.55 µM of carbachol (CCh) and when the plateau was achieved, NaHS (0.2, 0.4, 0.6, 0.8, 1.0 and 1.2 mM) was added cumulatively to the organ bath and left to achieve a new plateau for each concentration. To investigate the role of nitric oxide (NO), prostaglandin, cGMP productions and ATP-sensitive K^+^ channels, and cGMP production and β- adrenoceptors in the NaHS bronchodilatory activity, tissue preparations were incubated with L-NAME (300 μM), indomethacin (1 μM), methylene blue (10 μM), glibenclamide (1 μM), and propranolol (1 μM) respectively and the protocol of inducing contraction and applying cumulative concentrations of NaHS was repeated as described above. In these series of experiments each tissue preparation was used once for each experiment and the incubation period was 30 min for all applied antagonists and inhibitors. It is noteworthy that due to sensitivity differences of different segments of trachea being exposed to spasmogens, in all experiments, we used lower part of trachea for CCh and middle part for KCl protocols. Krebs-Henseleit solution was used to dissolve drugs, except indomethacin, which was dissolved in 100 mM of Na_2_CO_3_ solution. Chemicals were dissolved immediately before use and volumes added to bath were less than 5% of the organ bath volume. All dissection procedures were performed with extreme care to protect the epithelium from inadvertent damage. To examine the role of epithelium in the NaHS bronchodilatory effect, the epithelial cells were removed mechanically by rubbing the luminal surface of trachea with a fine cotton-tipped applicator (thinned teeth stick moisten in Krebs-Henseleit solution). In these protocols, tracheal preparations with epithelium intact and denuded were used from the same animals. At the end of some experiments, the tissue was blotted and weighed then the contraction forces induced by KCl or CCh (at plateau level) were calculated to express as g force/mg tissue weight for comparing the spasmogenic activity of these spasmogens. 

***Statistical analysis***

Tracheal contractions (at plateau) induced by CCh or KCl were assumed as 100% and the remaining contraction (%) after applying NaHS was calculated and results were presented as mean±SEM. The number of animals were used for each protocol was presented by n. Statistical comparisons (SPSS) were made by repeated measurement ANOVA, one sample t test and independent ample t test. Values of* P*< 0.05 were considered statistically significant. 

## Results

***Effect of NaHS on tracheal basal tone***

To investigate the effect of NaHS (alone) on tracheal basal tone, after equilibrium period and under 1.5 g resting tension, NaHS was added to the tissue bath. The tracheal basal tone was not altered by cumulative concentrations of NaHS (0.2-1.2 mM). 

***Effect of NaHS on KCl- and CCh-induced tracheal contraction***

**Figure 1:**
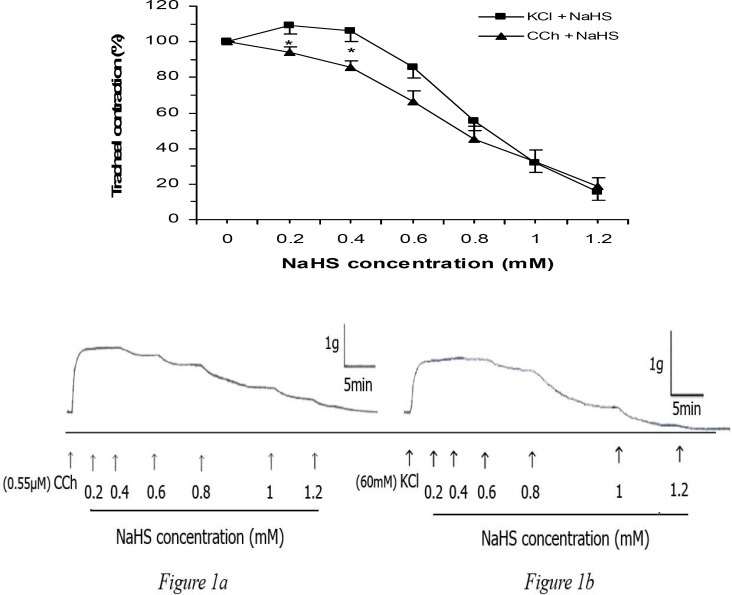
Effect of cumulative concentrations of NaHS on rat trachea contractions induced by KCl (60 mM, n= 8) and carbachol (CCh, 0.55 μM, n= 8). The relaxant effect of NaHS are concentration dependent for both spasmogens (ANOVA, *P*< 0.001) but they are not significantly different (two-ways ANOVA). Figures 1a and 1b are representing traces of these effects. The higher stimulus potency of CCh could be seen from comparison of vertical calibrations bars (1 g) of KCl and CCh results. Values are given as mean±SEM. * *P*< 0.05, KCl vs. CCh with the same concentrations of NaHS

Cumulative concentration of NaHS (0.2, 0.4, 0.6, 0.8, 1.0, and 1.2 mM) reduced KCl- and CCh-evoked tracheal contraction in a concentration-dependent manner and significantly (two-ways ANOVA, *P*< 0.001). These two concentration-response curves were not significantly different, although NaHS at 0.2 and 0.4 mM evoked greater (*P*< 0.05) relaxation on CCh-induced contraction (Figure 1). Low concentrations of NaHS (0.2 and 0.4 mM) augmented the KCl-induced contraction but not significantly different from the plateau of contraction induced by KCl. This NaHS stimulatory effect was not observed in the CCh precontracted tissue. In the presented typical traces of the NaHS relaxant effect on KCl- and CCh-induce tracheal contractions (Figures 1a and 1b), it can been seen that NaHS relaxant effect on CCh-induced contraction is more potent than on KCl-induced contraction with respect to 1 g calibration vertical bar.

***Role of epithelium on NaHS relaxant effect in precontracted trachea***

Removing the tracheal epithelium did not attenuate the NaHS bronchodilatory effect on tissue precontracted by KCl or CCh (Table 1). In the CCh-induced contraction, however, NaHS at higher concentrations (1 and 1.2 mM) induced more potent relaxation when the epithelium was denuded (*P*< 0.05 and *P*< 0.01 respectively). 

***Role of β-adrenoceptors and nitric oxide production in NaHS relaxant activity***

**Figure 2:**
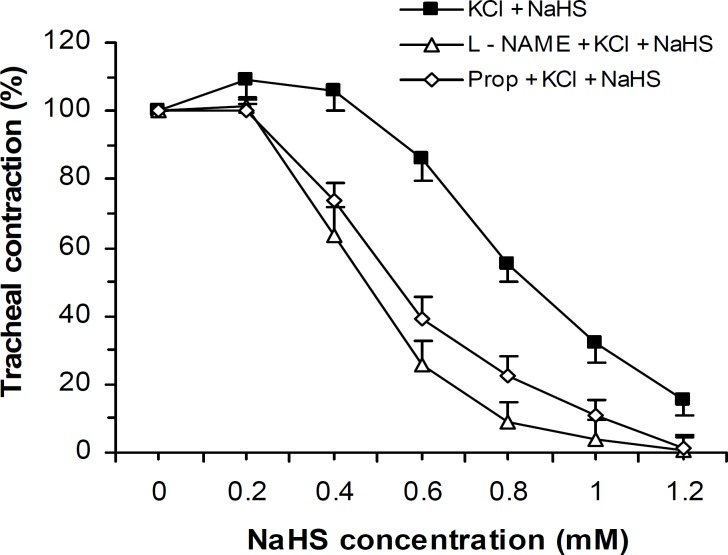
Effect of cumulative concentrations of NaHS on rat trachea contractions (mean±SEM) induced by KCl (60 mM, n= 8) before and after incubation with L-NAME (300 μM, n=8) and propranolol (Prop, 1 μM, n= 10). L-NAME and propranolol did not reduce the NaHS relaxant effect but rather potentiated this effect significantly (two-ways ANOVA, *P*< 0.001).

Bronchodilatory effect of NaHS on KCl-induced tracheal contraction was not reduced by tissue incubation with propranolol (β-adrenoceptor antagonist, 1 μM) and L-NAME (nitric oxide synthase inhibitor, 300 μM) but rather the relaxant activity of NaHS was augmented in the presence of these two agents (Two-ways ANOVA, *P*< 0.001) (Figure 2).

***Role of prostaglandins production in NaHS relaxant activity***

**Figure 3:**
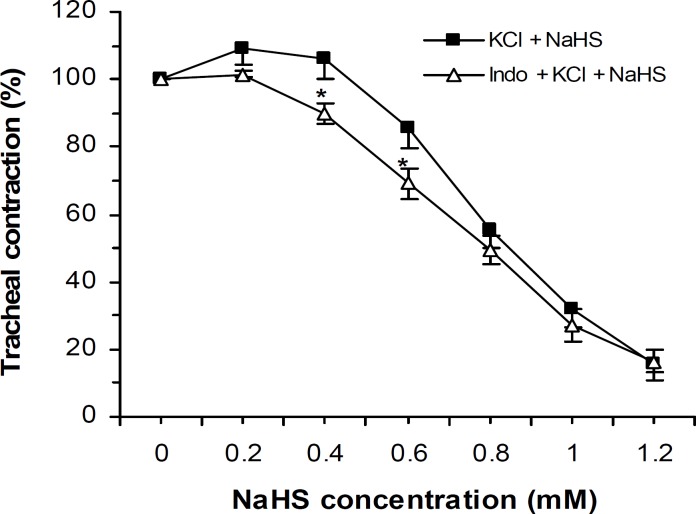
Effect of cumulative concentrations of NaHS on rat trachea contractions (mean±SEM) induced by KCl (60 mM, n=8) before and after incubation with indomethacin (Indo, 1 μM, n= 8) which are not significantly different (two-ways ANOVA). However, indomethacin potentiated the NaHS relaxant effect at 0.4 and 0.6 mM,* *P*< 0.05.

The NaHS bronchodilatory effect on KCl-induced trachea contraction was not attenuated by tissue incubation with 1 μM of indomethacin (nonselective cyclooxygenase inhibitor) (Figure 3).

***Effect of methylene blue and glibenclamide on NaHS relaxant activity***

**Figure 4:**
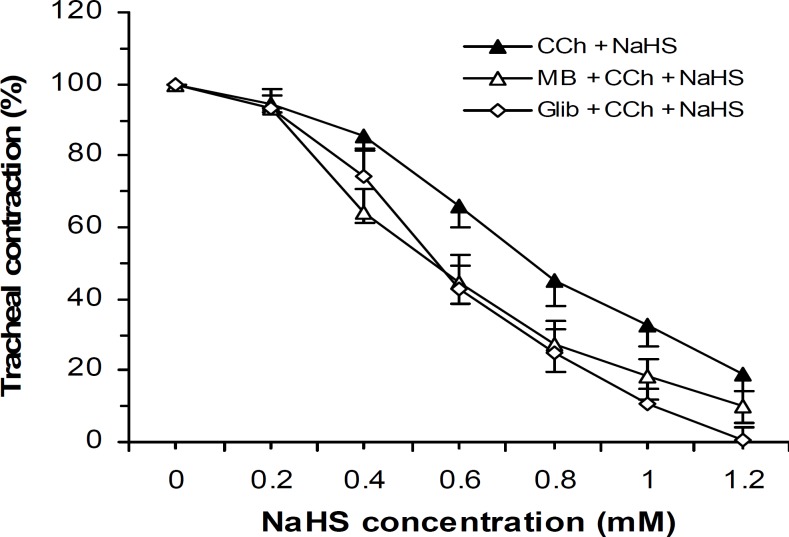
Effect of cumulative concentrations of NaHS on rat trachea contractions (mean±SEM) induced by CCh (0.55 μM) before (n=8) and after tissue incubation with glibenclamide (Glib, 1 μM, n=9) and methylene blue (MB, 10 μM, n=9). Glibenclamide and methylene blue were unable to attenuate the NaHS relaxant effect but rather augmented its effect (two-ways ANOVA, *P*< 0.05).

Separate tissues were incubated with methylene blue (a soluble guanylyl cyclase inhibitor, 10 μM) or glibenclamide (ATP-sensitive K^+^ channel blocker, 1 μM) and thereafter the tissue was contracted by CCh (0.55 μM). These inhibitor and antagonist were unable to reduce the NaHS relaxant effect but rather the relaxant effect was potentiated (Two-ways ANOVA, *P*< 0.05) by these agents (Figure 4).

## Discussion

The results of the present study demonstrate that NaHS as a H_2_S donor has a relaxant effect on rat trachea precontracted by KCl or CCh. The observed relaxing effect was not dependent on the integrity of airway epithelium.

**Table 1:** Effect (in %) of cumulative concentrations of NaHS on rat tracheal contractions (mean±SEM) induced by KCl (60 mM) and CCh (0.55 µM) in the presence of intact and denuded epithelium

		NaHS (mM)						
		0.0	0.2	0.4	0.6	0.8	1.0	1.2
KCl	Epi. Intact	100 (0)	108 (4.8)	106.1 (6.1)	85.9 (6.2)	55.3 (5.5)	32.1 (5.9)	15.7 (4.5)
	Epi. denuded	100 (0)	106 (2.0)	97.9 (5.2)	63.4 (12.1)	38.5 (10.1)	18.2 (7.8)	13.2 (7.9)
CCh	Epi. intact	100 (0)	94.3 (2.5)	85.5 (3.6)	66.2 (6.3)	45.4 (7.2)	32.8 (6.4)	18.9 (4.4)
	Epi. denuded	100 (0)	98.7 (0.7)	87.4 (4.8)	55.5 (9.4)	30.9 (5.0)	10.5 (3.6) *	5.7 (2.7) **

Data are presented as the mean ± standard error of mean (in parentheses) and n= 8 for each experiment. *P< 0.05, **P< 0.01 represent the significance of the intact in comparison to denuded epithelium (Epi.).

In addition, β-adrenoceptors, ATP-sensitive potassium channels, nitric oxide, prostaglandins, and cGMP were not involved. 

Dissolved NaHS quickly dissociates into Na^+ ^and HS^-^ ions which, in turn, react with H_2_O to give H_2_S and NaOH. However, it is reported that NaHS at high concentration (30-50 mM) increases Krebs pH solution only 0.1-0.2 U but the same elevation in pH was unable to alter tracheal tension (2). In our study the highest applied NaHS concentration was 1.2 mM, therefore, bath solution pH alteration could not be a factor to change the tissue contractility. The excitatory and inhibitory effects of H_2_S have been demonstrated (13, 14) however, in our study, NaHS did not induce any alteration in the tracheal basal tone.

Hydrogen sulfide induces spasmogenic and spasmolytic effects on different smooth muscle tissues (2, 12), therefore, these responses are apparently depends on the tissues and the species. On the other hand, the elevated endogenous H_2_S in COPD patients may show the physiological or pathological role of this gas in human but in our study, the study was based on the pharmacological concentrations. In addition, it is possible that H_2_S has been elevated in these patients to reduce airway resistance as a physiological response. 

In our study, the contractions evoked by KCl and CCh were sustained as long as they were present in the tissue bath solution as tested with inducing contraction by the spasmogens in the absence of NaHS for at least 60 min as reported for acetylcholine (Ach) (15). The observed NaHS relaxant effect, therefore, was not related to tissue fatigue. The calculated contraction force at plateau for CCh and KCl were 0.254±0.02 vs. 0.164±0.02 g/mg tissue wet weight respectively indicating that the CCh was more potent than KCl (*P*< 0.01) to evoke contraction. 

Potassium chloride causes contraction by a highly reproducible and relatively simple mechanism by activating the voltage dependent calcium channels that results in elevation of cytosolic free Ca^2+^, myosin light chain kinase activation and ultimately causing contraction. Hyperosmotic solutions reduce rat tracheal contractility (16) but, the maintained and sustained contraction induced by KCl (60 min without NaHS) indicated that the hyperosmolarity caused by applied KCl was not responsible for NaHS relaxant effect.

Carbachol induces tracheal contraction via muscarinic M_3_ receptors (17). Stimulation of M_3_ receptors activates phospholipase C and increases IP_3_ synthesis which in turn, promotes calcium release from sarcoplasmic reticulum and at the same time, Ca^2+^ influx from the extracellular space, causing elevation in the intracellular Ca^2+^ concentration which causes contraction (17). 

High potassium (more than 30 mM) is known to induce smooth muscle contractions via opening of voltage-dependent L-type calcium channels thus allowing influx of Ca^2+^ from extracellular space and a substance causing inhibition of high potassium-evoked contraction is considered as a calcium influx blocker (18). It seems therefore, that in KCl-induced contraction, NaHS inhibits Ca^2+^ influx, possibly by inactivation of the voltage dependent calcium channels. The NaHS relaxant effect in our study is consistent with other reports (19). On the other hand, CCh elevates intracellular calcium using two pathways (promoting Ca^2+^ influx and releasing Ca^2+^ from intracellular pools) therefore, it may be concluded that NaHS antagonizes both pathways (Ca^2+^ influx inhibition and M_3_ receptors inactivation) simultaneously. Although, the involvement of Ca^2+^ influx in the vasorelaxatory effect of NaHS has been also demonstrated (20). As mentioned in results section, CCh was more potent than KCl to evoke contraction (Figure 1a, 1b), and on the other hand, the more potency of NaHS on CCh-induced contraction in compare to KCl, could be results of blocking of these two pathways by NaHS. In our study, the NaHS relaxant effect at the final concentration (1 and 1.2 mM) for the applied spasmogens was similar indicating these two pathways were almost blocked by NaHS. 

After removing the epithelium, NaHS only at 1 and 1.2 mM evoked more potent (*P*< 0.05) relaxation on CCh-induced contraction but the alteration was not observed for KCl-induced contractions. However, it has been demonstrated that in guinea-pig trachea the contractile effect of CCh and KCl were unaffected by epithelium removal (21). On the other hand, the tracheal relaxation response was unaffected in the epithelial denuded trachea in rat (22) which is consistence with our results in which the NaHS relaxant effect was not reduced by epithelium removing.

The relaxatory effect of β-adrenoceptors in rat trachea has been reported (23) but in the present study, propranolol as a nonselective β-adrenoceptor antagonist did not attenuate the NaHS relaxant effect which ruled out the possible involvement of β-adrenoceptors in the observed effect. Prostaglandin E_2_ induces bronchodilation (24) which released from epithelium and causes airway relaxation.

In this study, however, tissue incubation with indomethacin as a nonselective cyclooxygenase inhibitor did not reduce the NaHS relaxant activity. Therefore, it seems that prostaglandins have not been involved in this activity. Furthermore it is documented that (NO) is a mediator of tracheal relaxation (25) and H_2_S enhances NO production (26). In the present study, however, the NO synthase inhibitor, L-NAME, was unable to reduce the NaHS relaxant effect but rather augmented this effect. It seems therefore, nitric oxide production is not implicated in the NaHS effect. Although, NO relaxes smooth muscle through guanylyl synthase activation to produce cGMP, but in our study methylene blue, as a soluble guanylyl synthase inhibitor, did not attenuate the NaHS relaxant effect, therefore, it seems that, cGMP production was not involved in NaHS relaxant effect and this result is consistent with H_2_S-induced vasorelaxation (20). 

However, if the H_2_S bronchodilatory effect was neither via β-adrenergic receptors nor NO, we expect to have no changes in the bronchodilatory effect but not augmentation in the presence of propranolol and L-NAME. But the reason for the observed augmentation in present study can not be explained and needs to be more investigated.

The vasorelaxant activity of H_2_S is caused by opening vascular smooth muscle cells ATP-sensitive potassium channels (27) which leads to membrane hyperpolarization, therefore, H_2_S may reduce Ca^2+^ influx and relaxes vascular tissue (28). Our results showed that the NaHS relaxant effect was not reduced by glibenclamide as a selective K_ATP_ channel opener which indicates that these channels were not involved. However, it has been shown that H_2_S-induced human corpus cavernosum smooth muscle relaxation (29) was inhibited by 150 µM of glibenclamide which is much higher than 1 µM that we used in our study. It is, therefore, possible that the concentration differences of applied glibenclamide caused the different results. 

In addition, cytochrome C oxidase in mitochondrium is one the intracellular target of H_2_S which inhibits mitochondrial respiration (30) In our study, after tissue exposing with NaHS, refreshing the bath solution returned tissue contractility to 88 and 94% (for CCh and KCl respectively) of their initial contractility, therefore, the applied NaHS should not inhibit trachea contraction by this mechanism, since refreshing bath solution can not remove NaHS from mitochondria media.

## Conclusions

The present results demonstrated that NaHS as a H_2_S donor attenuated the rat tracheal contractions induced by KCl and CCh. This effect was neither dependent on trachea epithelium nor NO, cGMP and prostaglandins production. Furthermore, β-adrenoceptors, and ATP-sensitive potassium channels were not involved. Our results supported the possible involvement of calcium influx and cholinergic receptors inactivation. Further research is definitely needed to explore the mechanism(s) of NaHS bronchodilatory activity and possible therapeutical property of H_2_S in obstructive pulmonary diseases.
